# Quality of Life in European Adults and Older with All-Over Pain: Relationship with Frequency of Moderate and Vigorous Physical Activity and Decision Prediction Models with Cross-Sectional Data

**DOI:** 10.3390/healthcare13101171

**Published:** 2025-05-17

**Authors:** Angel Denche-Zamorano, Daniel Collado-Mateo, Juan Manuel Franco-Garcia, José Carmelo Adsuar, Diana Salas-Gómez

**Affiliations:** 1 Promoting a Healthy Society Research Group (PHeSO), Faculty of Sports Sciences, University of Extremadura, 10003 Cáceres, Spain; denchezamorano@unex.es (A.D.-Z.); jadssal@unex.es (J.C.A.); diana.salas.gom@gmail.com (D.S.-G.); 2 Centre for Sport Studies, Rey Juan Carlos University, 28943 Madrid, Spain; 3 Health, Economy, Motricity and Education (HEME) Research Group, Faculty of Sport Sciences, University of Extremadura, 10003 Cáceres, Spain

**Keywords:** exercise, fibromyalgia, aging, health-related quality of life, machine learning

## Abstract

Background/Objectives: Quality of life (QoL) is negatively affected in people with all-over pain. Widespread pain has a negative impact on physical function, cognitive function, mental health and mood. Physical activity (PA) may help to improve the QoL in these people. This study aimed to assess the relationships between PA frequency (PAF) and QoL in middle-aged and older European people with all-over pain, in addition to developing and analyzing the performance of a classification and regression tree model (CRT) to predict QoL in this population. Methods: A cross-sectional study of 1025 middle-aged and older European individuals with all-over pain. Relationships between moderate and vigorous PAF and QoL were assessed. A predictive algorithm for QoL was developed using CRT analysis. A cross-validation study was conducted to assess the performance of the model. In addition, a multivariate linear regression model was developed to predict QoL and compare its performance with the CRT model. Results: Higher PAF and higher QoL were found to be related (*p* < 0.001). Specifically, the CRT found that depression, poor perceived health, and moderate physical activity once a week, rarely or never were the combinations of variables that predicted lower quality of life scores. Conversely, not having depression and performing moderate physical activity more than once a week predicted higher quality of life scores. The linear regression model performed better than the CRT model (R^2^ = 38% vs. R^2^ = 30%), and both identified depression, SPH, moderate PAF and education level as main predictors of QoL. Conclusions: PA on a regular basis could improve the QoL of people with all-over pain. Depressive symptoms, self-perceived health, PAF and educational level are predictors of QoL in this population. Our findings provide useful information for assessing QoL in people with all-over pain, offering an easy-to-interpret visual model with similar accuracy to traditional models.

## 1. Introduction

All-over pain or widespread pain (WP) is a diffuse pain [1], and is difficult to localize to a specific point of the body. In fact, it is a pain that spreads all over the body or a large part of it [2,3]. It has also been defined as pain appearing on the left and right sides of the body, above and below the waist, and in the axial skeleton [4,5]. WP can occur acutely with actual or potential tissue damage and can be caused by various factors such as injuries, infections, illnesses or significant physical or psychological stress [6,7]. Acute pain is usually short-term and tends to improve as the underlying cause is treated [8,9]. On the other hand, chronic pain is defined as pain that persists beyond the time of healing of an injury or the apparent reason for appearance, or recurs for longer than three months [10]. Chronic widespread pain (CWP) is a chronic condition that affects 10–15% of the general population, although some studies suggest that the prevalence may be as high as 24%, being even more prevalent in older people [4,11]. Its onset is usually due to multifactorial and complex causes, combining environmental, biological, social and psychological factors [1]. Although it can occur at any point in the life cycle, it is most commonly found in middle-aged and older people and is particularly prevalent in women [5,12]. In these populations, CWP may be due to musculoskeletal disorders, osteoporosis, arthritis or other pathologies [13,14], including fibromyalgia [15]. Fibromyalgia is one of the most severe clinical manifestations of CWP [3], being an early indicator of this pathology [5]. CWP is frequently associated with other somatic symptoms such as fatigue, psychological stress and concentration difficulties [4,16,17]. Consequently, widespread pain can negatively impact on the overall functionality, mobility, physical condition, mental health and, in general, the quality of life (QoL) of affected individuals [14,18,19].

QoL is a complex concept that refers to the overall well-being of people, combining objective and subjective aspects of life [20,21]. It includes everyone’s notion of their state of health, physical well-being, social and emotional state, considering their individual situation and living conditions [21]. Among several factors that can affect QoL, it is not only CWP as discussed above that can affect QoL [14,18,19]. In this sense, experiencing any type of pain, whether localized, acute, in different parts of the body, etc., also has a significant impact on QoL [14,18,19]. In addition, other factors or conditions, such as mental illness (e.g., depression and its symptoms), advanced age, obesity, smoking, sleep problems, cognitive difficulties, social isolation, low educational level, low social status or physical inactivity, have been identified as factors that negatively influence QoL [22,23,24,25,26,27,28,29]. In contrast, social support, emotional well-being, life satisfaction, higher educational level or physical activity (PA) have been identified, among others, as factors that can positively affect perceived QoL [30,31,32,33].

In this regard, PA, at both moderate and vigorous intensities, has been shown to be related to a higher QoL [33,34,35], with a lower presence of pain and a lower level of pain in people who experience it, as well as being inversely related to modifiable factors that negatively affect QoL [33,36,37]. For example, PA has been reported to be related to a lower presence of depressive symptoms, less social isolation, less obesity or lower prevalence of sleep problems and cognitive difficulties [38,39,40,41,42,43]. Conversely, PA is positively related to better self-perceived health (SPH), functional ability and physical fitness in both the general population and in people with pain [44,45,46,47,48].

Although it is widely recognized that pain has a negative impact on the QoL of those who suffer from it, and that a good QoL is an essential aspect of an individual’s life, perhaps due to the complexity of the concept of QoL, to the authors’ knowledge, there are no studies that attempt to predict the level of QoL of people with all-over pain using methodologies that help the professionals who treat them to make decisions. Therefore, the aims of the present study were: (1) to assess the relationships between the frequency of moderate and vigorous PA with QoL in the middle-aged and older European population with all-over pain; (2) to develop a classification and regression tree (CRT) to predict the QoL of this population according to the frequency of moderate and vigorous PA together with other socio-demographic variables and risk factors; this statistical method aims to obtain a hierarchical and graphical representation of interactions between the variables in the model and (3) To evaluate the performance of the CRT model in comparison to a multivariate linear regression model. The starting hypotheses were: (1) frequency of moderate and vigorous PA is related to quality of life in the middle-aged and older European population with all-over pain, while a higher frequency of PA will be related to higher quality of life; (2) frequency of moderate and vigorous PA will be among the variables of greatest relative importance in predicting quality of life in the middle-aged and older European population with all-over pain and (3) the classification and regression tree model will predict quality of life with equal or greater accuracy than the linear regression model, providing a graphical and hierarchical representation of variables and quality of life scores that is easier to interpret.

## 2. Materials and Methods

### 2.1. Design

A cross-sectional study was conducted. This study utilizes secondary data analysis from Wave 9 of the Survey of Health, Ageing and Retirement in Europe (SHARE). SHARE is a multidisciplinary survey designed to assess aging processes in adults across various European countries and Israel [49,50,51,52].

The SHARE survey is conducted biennially, employing representative samples from participating European nations and Israel. Similar to Wave 8, probability sampling was used, targeting households with at least one adult who spoke the official language of the country and was not residing abroad during the survey period. In addition to the longitudinal samples from previous waves and national refreshment samples included in Wave 8, Wave 9 also incorporates refreshment samples from batches not fielded before the suspension of Wave 8 fieldwork due to the COVID-19 outbreak in spring 2020. Detailed eligibility criteria are available in the SHARE Release Guide [53], which is publicly accessible on the SHARE-ERIC website (www.share-eric.eu/).

SHARE was initiated in 2004, and the project is under continuous evaluation. It has received ethical approval from the Ethics Committee of the University of Mannheim for Waves 1 to 4, and from the Ethics Board of the Max Planck Society for Wave 4 onwards and the continuation of the project [54].

Participants were interviewed through standardized face-to-face sessions by trained computer-assisted interviewers, conducted at the participant’s home, in nursing homes, or in other suitable environments. Consenting participants underwent a “main interview” encompassing various sections, including demographics and physical health. The comprehensive survey methodology details can be found in the methodology documentation [50].

Wave 9 of the SHARE survey was conducted between October 2021 and September 2022.

### 2.2. Sample

The initial sample consisted of 69,447 participants. The inclusion criteria established for the present study were as follows: (1) experiencing pain; (2) for the question about the location of the pain, those who reported having all-over pain were selected; (3) individuals between 50 and 80 years of age; (4) providing an answer to the question about QoL and (5) having data on the frequency of PA (moderate or vigorous).

**Figure 1:**
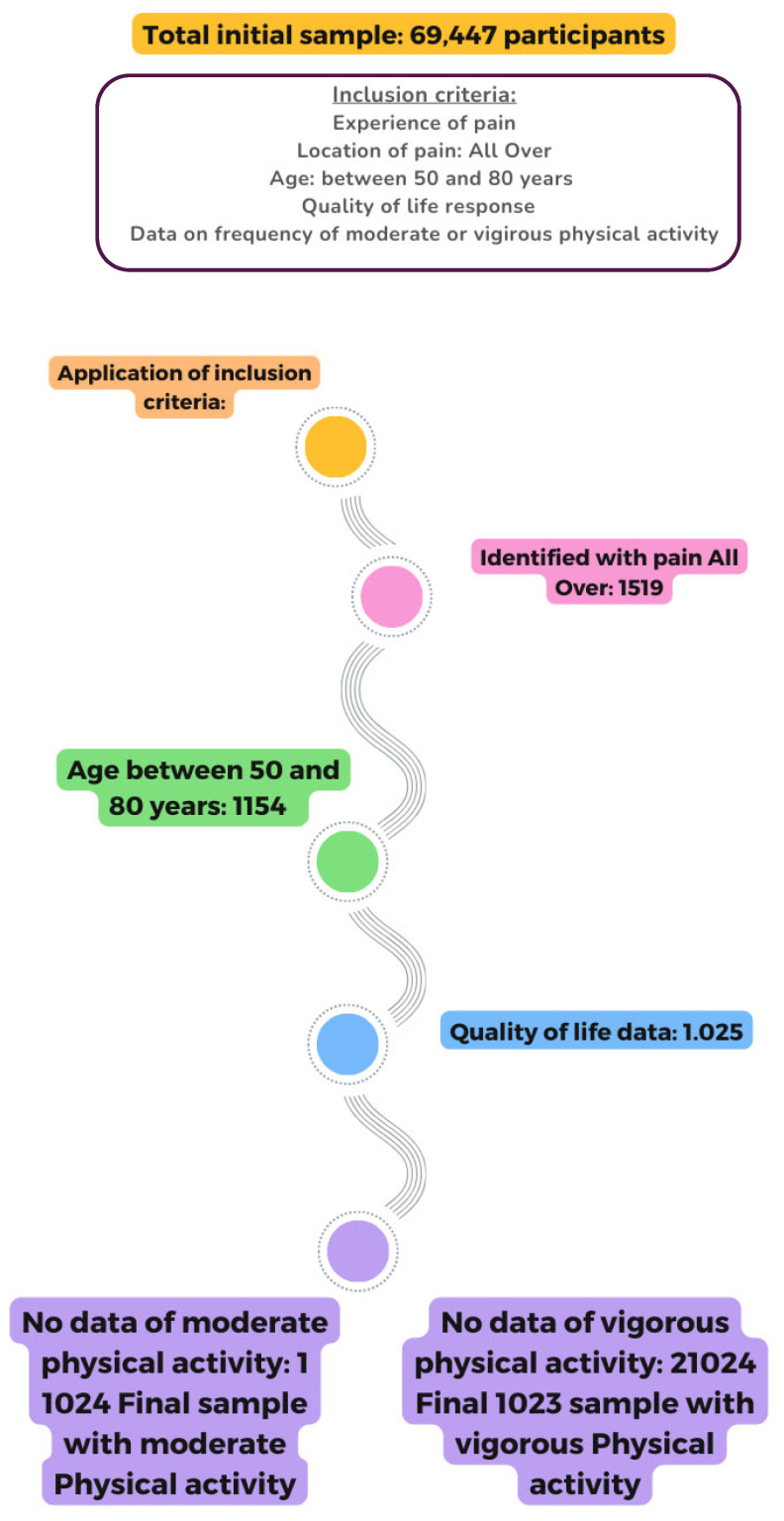
Flowchart of the sample selection process.

After applying the criteria, 1519 people were identified as having all-over pain. Among them, 1154 were between 50 and 80 years old, and 1025 had provided data on QoL. Additionally, 1 person did not report the frequency of moderate PA, and 2 did not report the frequency of vigorous PA. The final sample consisted of the following: (a) 1024 participants with all-over pain, aged 50–80 years, who reported their level of QoL and their frequency of moderate PA and (b) 1023 participants with all-over pain, aged 50–80 years, who reported their level of QoL and their frequency of vigorous PA (Figure 1)

### 2.3. Variables

Data sources are available at https://share-eric.eu/data/data-access, accessed on 7 April 2025. After gaining access to the data, the data were downloaded, and the following variables were extracted.

#### 2.3.1. Demographic and Predictor Variables

*Age* (Years), *Sex* (Men or Women), *Height* (cm) and *Weight* (kg) were recorded.

*Body mass index* (BMI, kg/m2) was categorized as follows: “Underweight” (BMI < 18.5 kg/m^2^), “Normal” weight (BMI ≥ 18.5 and < 25 kg/m^2^), “Overweight” (BMI ≥ 25 and <30 kg/m^2^)) and with “Obesity” (BMI ≥ 30 kg/m^2^).

*Educational level* was classified based on the International Standard Classification of Education (ISCED)–97) as follows: “None or pre-primary”; “Primary”; “Lower secondary”; “Upper secondary”; “Post-secondary non-tertiary”; “First stage of tertiary”; “Second stage of tertiary education” and “Still in school” [55].

*Level of pain:* participants were asked to report the intensity of pain as “Mild”, “Moderate” or “High”.

*Smoke at the present:* the following question was asked: Do you currently smoke? Possible answers were “Yes” or “No”.

*Self-perceptive health (SPH):* participants were asked to say how they would describe their general health with the following options: “Excellent”, “Very good”, “Good”, “Fair” and “Poor”. To the present study, the options “Excellent” and “Very good” were combined, leaving 4 possible responses.

*Depression Scale:* The assessment of self-reported depressive symptoms in this study was carried out using the EURO-D scale, developed by a European consortium. This scale consists of 12 items measuring various aspects of depression: depression, pessimism, suicidal thoughts, guilt, sleep quality, interest, irritability, appetite, fatigue, concentration (on reading or entertainment), enjoyment and tendency to cry. Each item assesses the self-reported presence of a specific symptom. The scale has a score range from 0 to 12, where a higher number of symptoms indicates a higher score and, consequently, a higher degree of depression. The EURO-D scale has shown good correlation with other recognized health measures, and its validity has been confirmed by several studies [56].

Responses were clustered into the following categories, with scores from 0 to 3 indicating “No” depression and scores of 4 or higher indicating “Yes” depression [57].

*Frequency of Activities requiring a moderate level of energy (Moderate PAF)*: participants were asked the following question: How often do you carry out activities requiring a moderate level of energy such as gardening, cleaning the car, or doing a walk? The possible answers were: “More than once a week”, “Once a week”, “One to three times a month” and “Hardly ever, or Never”. *Frequency of Sports or activities that are vigorous (Vigorous PAF):* participants were asked the following question: How often do you do vigorous physical activities such as sports, heavy housework, or a job that involves physical labor? The possible answers were: “More than once a week”, “Once a week”, “One to three times a month” and “Hardly ever, or Never”.

#### 2.3.2. Dependent Variables

*Quality of Life (QoL):* To assess QoL and well-being, the Control, Autonomy, Pleasure and Self-Realization (CASP–12) scale, a shortened version of the original CASP-19 scale, was used. This scale was specifically designed for use in the SHARE study (CASP-12v.1) [58,59,60]. The scale identifies specific aspects of QoL in aging and comprises four areas: control, pleasure, autonomy and self-realization.

The scale items, assessed on a 4-point Likert scale, measure the frequency with which certain feelings and situations are experienced (3 items for each domain). The range of scores is from 12 to 48 points, where higher scores indicate a higher QoL. The data for this variable was collected in a variable called *CASP Index for QoL*. Furthermore, QoL can be grouped into the following categories (QoL Categorical): “Low” (<35 points), “Moderate” (35–37 points), “High” (38–39 points) and “Very high” (>39 points) [61]. Analysis of the psychometric properties of the scale showed Cronbach’s alpha of 0.81.

### 2.4. Statistical Analysis

The normality assumption was checked using the Kolmogorov–Smirnov test (*p* > 0.05). For the descriptive analyses of the sample, the median and interquartile range were used for continuous variables. Frequencies and percentages were used for categorical variables. The Kruskal–Wallis test was used to assess the differences in the CASP for QoL and well-being score according to the frequency of moderate PA. The same test was applied for vigorous PA. In both cases, post hoc analyses (two-to-two comparisons) were performed using the Mann–Whitney U test. The magnitude of the differences was quantified through Hedges’ g (*g*) as effect size index according to the following interpretation: trivial (*g* < 0.2), small (0.2 ≤ *g* < 0.5), moderate (0.5 ≤ *g* < 0.8) and large (*g* ≥ 0.8) [62].

In addition, to assess the association between QoL (in categories) and frequency of PA (moderate and vigorous), Pearson’s Chi-square test and post hoc z-analyses of differences of proportions were used. The strength of the associations was quantified using Cramer’s V coefficients according to the following interpretation: insignificant (0.0–0.10), weak (0.10–0.20), moderate (0.20–0.40), relatively strong (0.40–0.60), strong (0.60–0.80) and very strong (0.80–1.00) [63].

A Classification and Regression Tree (CRT) analysis was used to estimate participants’ QoL as a function of SPH, BMI, depression, sex, age, level of pain, education level, current smoking status, frequency of vigorous sports or activities, and activities requiring a moderate level of energy. The technique creates a decision tree using automatic stepwise variable selection to identify mutually exhaustive and mutually exclusive subgroups of a population [64]. The CRT method is a robust tree-based classification and prediction approach that works well with large samples, allowing the division of the sample into different subgroups (nodes) according to the presumed impact of the variables in the model. Additionally, this statistical procedure provides a useful visualization of the impact of each independent variable in the form of a tree model. Each root splitting node creates two subgroups, which in turn split into two further subgroups. The tree continues to grow until the stopping criteria are met. Compared to other complex modeling techniques, CRT requires relatively small samples of at least 10 events per variable to achieve reasonable predictive modeling with stable performance [65].

The following statistical specifications were applied in the present study:

- Dependent variable in the model was QoL and well-being (CASP Score).
- Independent variables in the models were SPH, BMI, depression, sex, age, level of pain, education level, smoke at the present time, frequency of sports or activities that are vigorous and frequency of activities requiring a moderate level of energy.
- The significance level was set at 0.05.
- The measure of improvement could not be less than 0.0001, indicating modest differences between nodes (i.e., higher values produce trees with a reduced number of nodes) [66].
- The iteration interval was 50–100 (maximum–minimum) to obtain a balanced tree with a useful number of nodes.
- Missing independent values were excluded from the process. An automatic number of surrogates (one less than the independent variables) was used. This was used for case classification when there are missing data on the independent variable in the tree.
- Furthermore, 10-fold cross-validation was performed to test the stability of the decision tree [67]. The dataset is divided into ten randomly selected and approximately equal parts, each of which maintains a similar distribution of the data. The first nine parts of the data (90%) are used to construct the largest possible tree, and the remaining 10% are used to obtain initial estimates of the error rate of the selected sub-tree. The process is repeated 10 times using different combinations of the remaining 9 subsets of data and a different 1/10 data subset to test the resulting tree. The cross-validation process reveals the risk value for the 10 test samples [68].
- The absolute and normalized significance of each independent variable in the model was requested. A bar chart was constructed to visualize the importance of the variables in the model.

First, the tree was made without limiting the maximum depth, which, in the case of CRT, is 5. Subsequently, to avoid subclassification and to facilitate the visualization and interpretation of the tree, growth was limited to four levels of depth. The risk estimate and standard error of both model trees were obtained, which helped to select the model to be presented.

For scale dependent variables, the risk estimate is a measure of the within-node variance and is used as a model fit criterion. Lower values indicate a better model. The following equation was applied to calculate the model fit [69]:

$$S^{2}\;e\;=\frac{Risk\;value}{S^{2}\;y}$$

where,

S^2^ e = Error variance or proportion of variance due to error.

Risk value = Within-node variance.

S^2^ y= Variance of the dependent variable or of the root node or standard deviation of the root node squared.

The variance of the dependent variable explained by the model (S^2^ ×) or explained variance is

$$S^{2}\;x\;=\;(1-S^{2}\;e)\times 100$$

Finally, a multiple linear regression was performed with the same dependent variable and the same predictors. The coefficient of determination (R2) was obtained as a measure of model fit and the standard error of estimation.

Finally, the CRT models and the regression model were compared. For this purpose, the results in terms of risk estimation, standard error and model performance (R2) were compared between the two models. For all analyses, a significance level of less than 0.05 was assumed as statistically significant, and the IBM SPSS Statistical v.27 was used.

## 3. Results

### 3.1. Descriptive

The results found in the descriptive analysis to characterize the sample were presented in Table S1. The median values and the interquartile range (IQR) of the continuous variables (age, height, weight, CASP Index for QoL and Depression Scale) and the absolute and relative frequencies of the categorical variables (sex, BMI, education level, level of pain, smoke at the present time, depression, SPH, moderate PAF, vigorous PAF and QoL Categorical) were presented.

The median age of the sample was 62 (15) years. In addition, they had a CASP Index for QoL of 33 (9) and a Depression Scale of 4 (14). As main characteristics, 73% of the sample were female, 67% were overweight or obese, 88% of the sample had moderate to severe pain, 60% had depression, 75% perceived their health as fair or poor and 59% of the sample had a low QoL. In addition, 53% and 58% of the sample reported moderate or vigorous PA, respectively, between hardly ever or never.

### 3.2. Quality of Life in People with All-Over Pain According to the Frequency of PA

The CASP Indexes for QoL were found to be related to the moderate PAF (*p* < 0.001) and to the vigorous PAF (*p* < 0.001). In relation to the moderate PAF, the people with the worst QoL were those who reported doing moderate PA between hardly ever, or never, while the best QoL was reported by those who reported doing moderate PA more than once a week (30 vs. 36, *p* < 0.001, *g’* = 0.794). The same was found in relation to vigorous PAF, the best QoL was reported by the group who performed such activities more than once a week and the worst was reported by those who reported performing it hardly ever, or never (37 vs. 32, *p* < 0.001, *g’* = 0.581). These findings are shown in Table 1.

### 3.3. Association Between Quality of Life (Low, Moderate, High and Very High) and Physical Activity Frequency

The QoL level (QoL Categorical) was found to be related to the moderate PAF (X2 = 97.6, df = 9, *p* < 0.001, V = 0.178) and vigorous PAF (X2 = 56.2, df = 9, *p* < 0.001, V = 0.135). Table S2 shows that 77.8% of people with Very High QoL performed moderate PA more than once a week. In contrast, 65.2% of people with Low QoL reported performing vigorous PA hardly ever, or never.

**Figure 2:**
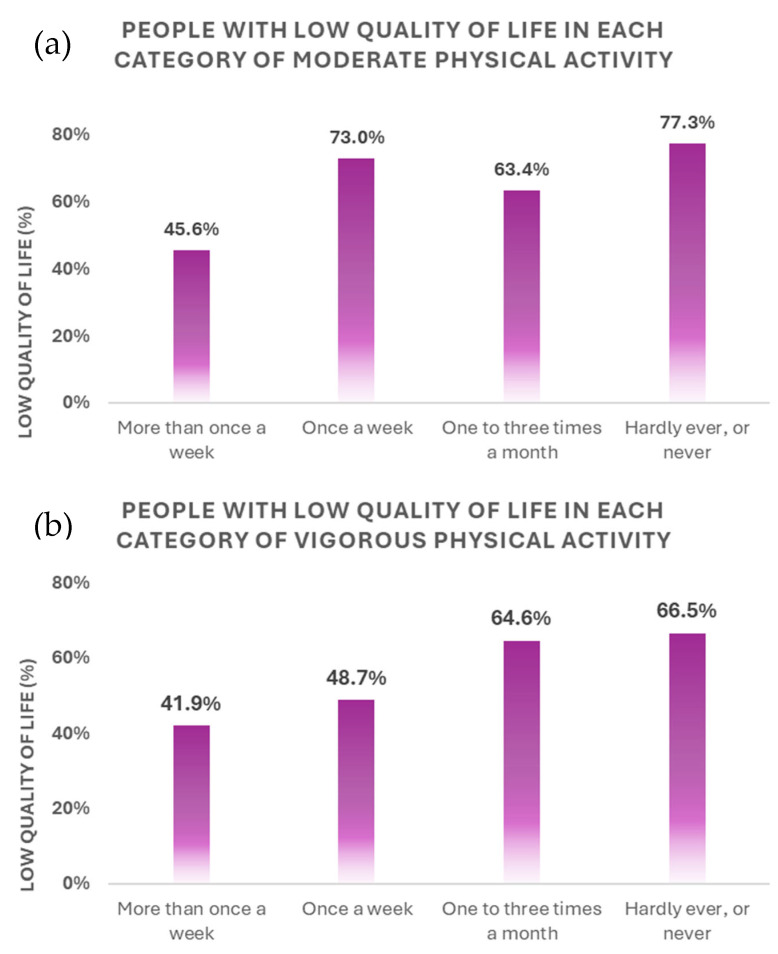
Low quality of life according to the frequency of (**a**) moderate and (**b**) vigorous Physical Activity. Differences were obtained between doing moderate intensity exercise more than once a week with the following frequencies: doing it once a week, doing it once or three times a month and doing it hardly ever or never (*p* < 0.001). Differences were obtained between doing vigorous intensity exercise more than once a week with the following frequencies: doing it once or three times a month and doing it hardly ever or never (*p* < 0.001). There were also differences between doing it once a week and doing it rarely or never (*p* < 0.001).

Complementarily, it was found that the lowest proportions of Low QoL were reported by those who engaged in moderate (45.6%) or vigorous (41.9%) PA more than once a week, while the highest proportions were reported by those who engaged in moderate PA hardly ever, or never (77.3%). Specifically, a higher prevalence of low QoL was found in those who never engaged in moderate PA (77.3% vs. 45.6%, *p* < 0.001) than in those who engaged in moderate PA more than once a week, as was the case for the vigorous PAF (65.5% vs. 41.9%, *p* < 0.001). Figure 2 shows the percentage of people with low QoL in each category of moderate and vigorous PAF.

### 3.4. CASP Index for QoL Prediction Model Using a Classification and Regression Tree

**Figure 3:**
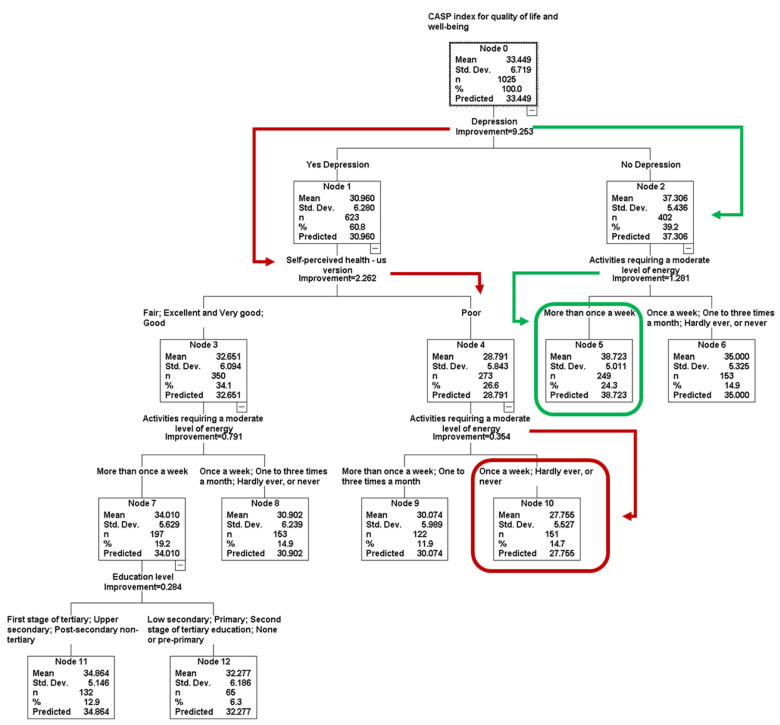
Classification and Regression Tree model for CASP Index for Quality of Life. The red lines and circle indicate the combination of conditions and the terminal node with the lower mean of CASP. The green lines and circle indicate the combination of conditions and the terminal node with the higher CASP. N (number); Std. Dev. (standard deviation); % (percentage).

The final decision-tree model for predicting the CASP Index for QoL (with limited depth) includes four levels and seven node terminals (Figure 3).

The worst QoL was found in Node 10 (CASP Index for QoL = 27.7; SD = 5.5), this node was formed by people who presented depression, poor SPH and who performed moderate PA once a week, hardly ever, or never. In contrast, the highest QoL was found in Node 5 (CASP Index for QoL = 38.7; SD = 5.0), people without depression reported moderate PA more than once a week. Table 2 shows the gain summary of the model.

Depression appeared as the most important predictor in the model (100%) along with nine other predictors (Table 3). This model had an estimation error of 31.7 (SE = 1.4) and explained 30% of the variance of the CASP Index for QoL (R2). This model presented a better estimation than the first model (without limiting the depth), which presented five depth levels, eight terminal nodes, an error of 32.8 (SE = 1.4) and explained 27% of the variance (R2). The without depth limitation model was presented in Figure S1.

### 3.5. Linear Regression Model for QoL

The multivariate linear regression analysis to predict the CASP Index for QoL presented four factors as predictors: SPH, depression, moderate PAF and education level. This model explained 38% of the variance (R2). Table 4 shows the linear regression model.

### 3.6. Comparison of Model Performance

Both CRTs showed very similar performances, with the four-level depth limited model performing slightly better (R^2^ = 30%) than the model without depth limitation (R^2^ = 27%). Both models showed a lower performance than the linear regression model (R^2^ = 38%). However, both models identified the same main predictor variables and in the same order of importance: depression, SPH, moderate PAF and education level.

## 4. Discussion

The current study aimed to evaluate the associations between the frequency of moderate and vigorous PA and quality of life in individuals with all-over pain, as well as to develop a simplified classification and regression tree (CRT) algorithm to predict quality of life in this population based on sociodemographic predictors (age, sex and educational level) and other predictors (BMI, self-perceived health, frequency of moderate and vigorous PA, depression, smoking and pain level).

The main finding was that both moderate and vigorous PA frequencies were found to be significantly associated with QoL in the European population with all-over pain (*p* < 0.001). These findings support our first hypothesis and are in line with the previous research demonstrating positive dose–response relationships between PA and health-related QoL across various populations [70,71,72].

PA is recommended in almost any long-term condition such as cancer, diabetes, cardiac disease or COPD since it is related to reduced risk of mortality [73]. However, dose–response effects of PA are still controversial. In the current study, participants engaging in moderate or vigorous PA more than once weekly exhibited significantly higher QoL scores (36 and 37 points, respectively) compared to those who rarely or never engaged in such activities (30 and 32 points, *p* < 0.001). This is consistent with evidence showing that moderate to vigorous PA correlates significantly with global QoL and pain levels in people with chronic pain [74]. Similarly, greater prevalence rates of poor QoL were observed among individuals who never engaged in moderate PA (77.3% vs. 45.6%, *p* < 0.001) or vigorous PA (65.5% vs. 41.9%, *p* < 0.001). Although findings from this study emphasize the importance of the frequency of moderate and vigorous PA, previous research suggests that, in the long term, only high levels of PA led to reduced risk of suffering from musculoskeletal pain compared to a sedentary lifestyle [75].

The CRT model revealed that depression is the most relevant predictor, with individuals experiencing depression scoring significantly lower on QoL measures compared to those without depression (31.0 vs. 37.3). The notion that psychological variables may be even more important than pain levels to determine the QoL of people suffering from pain is widely known. In this regard, social functioning, vitality, mental health and general health conditioned by thoughts about pain [76], make PA a key strategy to reduce depressive symptoms, especially among those with the highest levels of pain [77]. Consequently, depression should be prioritized as the first factor for assessing QoL in individuals with all-over pain. The model also highlighted self-perceived health and moderate PA frequency as critical variables. The lowest QoL scores were observed in individuals experiencing depression, perceiving their health as poor, and engaging in moderate PA less than once per week (27.8 points), which was 11 points lower than those without depression who engaged in moderate PA more than once per week (38.7 points). Educational level also emerged as an important variable within the CRT model, ranking fourth in importance, with vigorous PA frequency placed below it. Thus, our second hypothesis was only partially supported.

The linear regression model identified the same predictors with identical hierarchical importance but demonstrated superior performance compared to the CRT model (adjusted R²: 38% vs. 30%). Depression remained the most relevant factor affecting QoL scores, followed by poorer self-perceived health, lower moderate PA frequency and lower educational levels. Although our third hypothesis was not fulfilled due to the linear regression model’s better fit, both models are useful since linear regression may offer greater precision in predicting QoL while the CRT model may provide an easily interpretable framework that could serve as an initial assessment tool. Thus, the two models may offer complementary insights when evaluating complex QoL relationships. Although no specific studies were found that directly compare linear regression models and decision trees in predicting the quality of life in people with widespread pain, there is research that discusses the advantages and disadvantages of traditional statistical models versus machine learning in healthcare contexts. In this respect, our results are in line with the findings of Makino et al. [78], who developed a simplified decision tree algorithm to predict falls among community-dwelling older adults. In their study, they report that decision tree models, despite sometimes having lower predictive accuracy compared to logistic regression, offer intuitive and easily interpretable frameworks that can be valuable in clinical settings.

Although the reduction in pain will lead to increased HRQoL and reduced healthcare resource use [79], the identification of depression as the primary predictor of QoL in all-over pain patients has significant clinical implications, suggesting that psychological assessment should be prioritized when evaluating these individuals. Our findings suggest that interventions targeting both psychological wellbeing and PA promotion could potentially yield the greatest improvements in QoL for all-over pain patients. Even though our regression model explained a substantial portion of QoL variance (38%), additional factors may be involved. For instance, having low wealth, being female, and being overweight or obese have been identified as potential risk factors for chronic pain [75], but our results indicated that BMI and gender only achieved a normalized importance of 2.4% and were not included in the model to predict HRQoL.

This study has several important limitations that should be considered when evaluating and interpreting our findings. First, the cross-sectional design of this study, based on survey data, inherently carries limitations typical of this type of research. Although cross-sectional studies are valuable for identifying associations, assessing risk factors, determining prevalence rates, and generating hypotheses for future research, the extraction of causal and long-term conclusions is limited. Another significant limitation is the lack of objective data for some variables, as they were based on subjective assessments and/or self-reported information provided by participants. For instance, BMI was not objectively assessed, which could have altered the results of the models. Consequently, future studies employing alternative designs and incorporating objective measures are recommended to confirm the causal relationships underlying the associations identified in this study. Such research would also enable the development of appropriate dose–response analyses and effective prevention and intervention programs aimed at improving QoL in individuals with all-over pain.

## 5. Conclusions

The frequency of moderate and vigorous PA is related to quality of life in the European population with all-over pain. For both intensities of PA, a frequency of several days per week was associated with a higher quality of life than those who did no or occasional PA.

Both the CRT model and the linear regression model identified depression as the main predictor of quality of life along with self-perceived health, frequency of moderate PA and educational level as other important predictors. Although both identified and ranked predictors in the same way, the linear regression model had a better fit than the CRT model in predicting quality of life, although the CRT model provided a simplified visual predictive model that may facilitate the interpretation of the model.

This study allows us to stratify the population with all-over pain according to the expected quality of life based on predictor variables, providing useful information for professionals interested in the prevention and intervention of quality of life in people with all-over pain.

## Figures and Tables

**Table 1:** Quality of life according to the frequency of vigorous and moderate physical activity in people with All-over pain.

	CASP Index for QoL							
Moderate Physical Activity Frequency	Median	(IQR)	KW	df	*p*-Value	Post hoc MW	*p*-Value ^	g ’Hedges
More than once a week (1)	36	(9)	113.0	3	<0.001			
						1 vs. 2	<0.001 **	0.559
Once a week (2)	32	(8)				1 vs. 3	0.001 **	0.386
						1 vs. 4	<0.001 **	0.794
One to three times a month (3)	33	(8)				2 vs. 3	0.228	−0.172
						2 vs. 4	0.012 *	0.253
Hardly ever, or never (4)	30	(9)				3 vs. 4	0.002 **	0.406
		
**Vigorous physical activity frequency**								
More than once a week (1)	37	(8)	90.0	3	<0.001			
						1 vs. 2	0.600	0.182
Once a week (2)	36	(9)				1 vs. 3	0.002 **	0.384
						1 vs. 4	<0.001 **	0.581
One to three times a month (3)	33	(9)				2 vs. 3	0.172	0.199
						2 vs. 4	<0.001 **	0.403
Hardly ever, or never (4)	32	(9)				3 vs. 4	0.095	0.214
		

CASP (Control, Autonomy, Pleasure and Self-Realization (CASP-12) scale); QoL (Quality of Life); IQR (Interquartile Range); KW (Kruskal–Wallis test); df (Degrees of freedom); MW (Mann–Whitney U test for two independent samples); ^ (*p*-value for two-to-two comparisons); * (*p* < 0.05); ** (*p* < 0.01).

**Table 2:** Gain Summary for terminal Nodes.

Node	*n*	Percent	Mean
No Depression; Do More than a Week Moderate Physical Activity	249	24.3%	38.7
No Depression; Do Once a week, One to three times a month, Hardly ever, or never moderate Physical Activity	153	14.9%	35.0
Depression; SPH: Excellent or very good or good or fair; Do More than a week moderate Physical Activity; Educational Levell: Upper secondary or Post-secondary non-tertiary or First stage of tertiary	132	12.9%	34.9
Depression; SPH Excellent or very good or good or fair; Do More than a week moderate Physical Activity; None or pre-primary or Primary or Low secondary or Second stage of tertiary education	65	6.3%	32.3
Depression; SPH Excellent or very good or good or fair; Do Once a week, One to three times a month, Hardly ever, or never moderate Physical Activity	153	14.9%	30.9
Depression; SPH: Poor; Do More than a week or One to three times a month moderate Physical Activity	122	11.9%	30.1
**Depression; SPH: Poor; Do Once a week or Hardly ever, or never moderate Physical Activity**	**151**	**14.7%**	**27.8**

Growing method: CRT; dependent variable: CASP index for Quality of Life; mean: represents the score of CASP Index for QoL; SPH (self-perceptive health); n (participants).

**Table 3:** Cross-validation results of CRT models: estimations (standard errors of classification) and importance of the independent variables for CASP Index for Quality of Life in people with all-over pain.

Independent Variable	Importance	Normalized Importance	
Depression	9.253	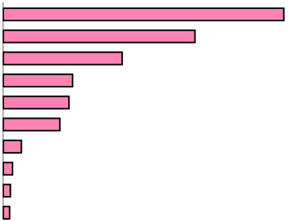	100.0%
Self-perceived health	6.286		67.9%
Moderate Physical Activity Frequency	3.945		42.6%
Education level	2.237		24.2%
Vigorous Physical Activity Frequency	2.181		23.6%
Level of pain	1.869		20.2%
Age	0.369		4.0%
Sex	0.223		2.4%
BMI	0.220		2.4%
Smoke at the present time	0.217		2.3%
		Estimation	(SE)
Cross-Validation (5 levels; 8 nodes terminals)		32.8	(1.4)
Model performance (R^2^)		27%	
Cross-Validation (4 levels; 7 nodes terminals)		31.7	(1.4)
Model performance (R^2^)		30%	

BMI (body mass index); SE (standard error).

**Table 4:** Linear regression model for CASP Index for Quality of Life.

						95% CI for B	
	B	St.	Beta	t	Sig.	Lower	Upper
(Constante)	44.376	1.301		34.110	<0.001 ***	41.818	46.933
Depression	−4.543	0.589	−0.333	−7.719	<0.001 ***	−5.700	−3.386
Self-perceived health	−2.493	0.362	−0.312	−6.886	<0.001 ***	−3.204	−1.781
Moderate Physical Activity Frequency	−0.772	0.234	−0.137	−3.306	0.001 **	−1.231	−0.313
Education level	0.601	0.209	0.117	2.883	0.004 **	0.191	1.012
Adjusted R^2^ (error of estimation)	38%	5.298					

B (unstandardized coefficient beta); St. (standard); Beta (standardized coefficient beta); t (t statistic); Sig. (statistical significance); CI: confidence interval; R^2^ (coefficient of determination); ** (*p* < 0.01); *** (*p* < 0.001).

## Data Availability

The original contributions presented in the study are included in the article/Supplementary Material; further inquiries can be directed to the corresponding authors.

## Supplementary Materials

The following supporting information can be downloaded at: https://www.mdpi.com/article/10.3390/healthcare13101171/s1, Figure S1: Classification and Regression Tree model for CASP Index for Quality of Life (Model without depth limitation); Table S1: Descriptive analysis of people with all-over pain.; Table S2: Prevalence of Quality of Life according to moderate and vigorous PA frequency.
