# Supplementary material for: Quality of Life in European Adults and Older with All-Over Pain: Relationship with Frequency of Moderate and Vigorous Physical Activity and Decision Prediction Models with Cross-Sectional Data
Source: Healthcare (Basel). 2025 May 17;13(10):1171. doi: 10.3390/healthcare13101171 (PMC12111565; doi:10.3390/healthcare13101171)
Supplement: Supplementary file 1 [file healthcare-13-01171-s001.zip › healthcare-3627459-supplementary.pdf]

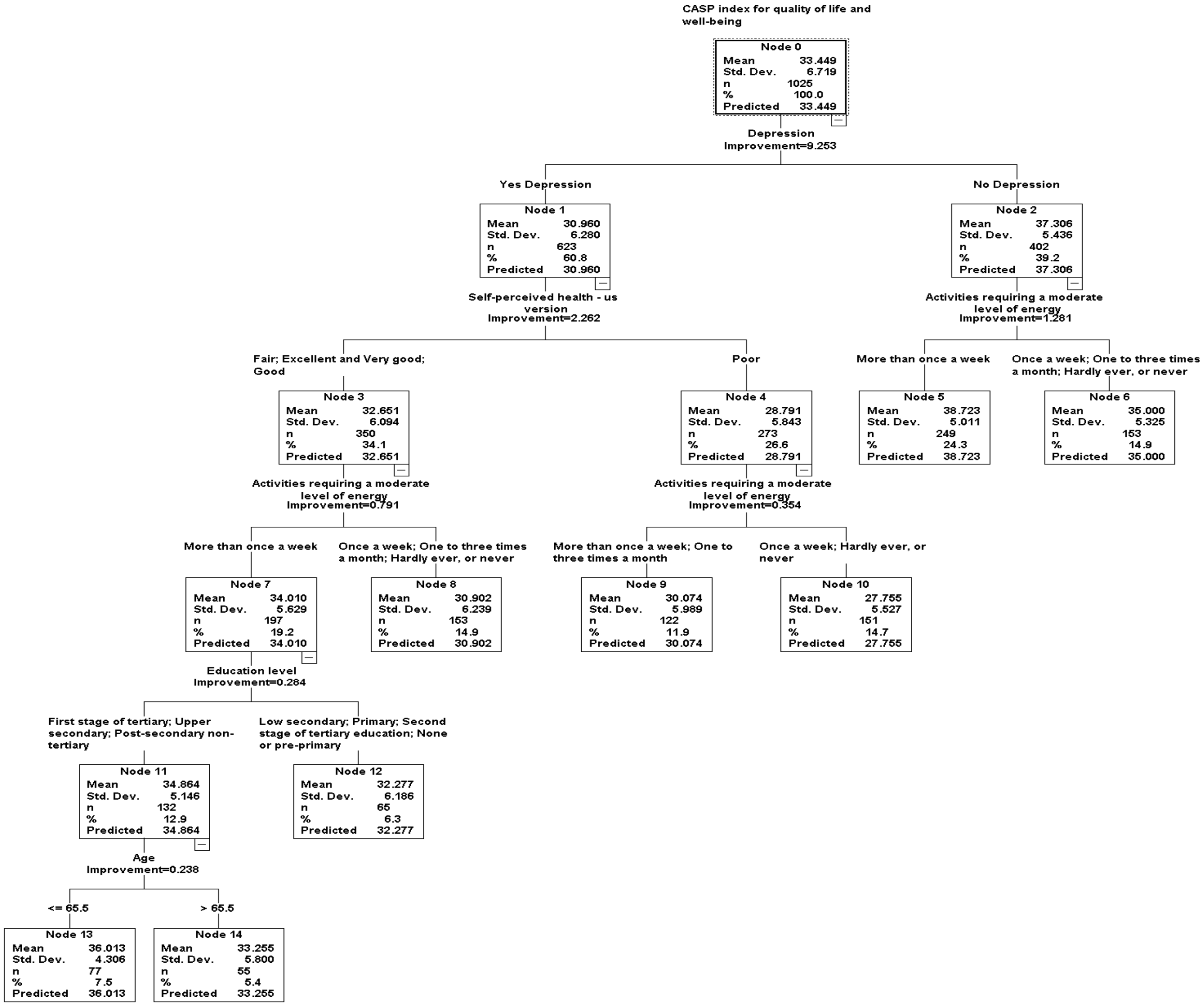
CASP index for quality of life and well-being
Node 0
Mean 33.449
Std. Dev. 6.719
n 1025
% 100.0
Predicted 33.449
Depression
Improvement=9.253
Yes Depression
Node 1
Mean 30.960
Std. Dev. 6.280
n 623
% 60.8
Predicted 30.960
No Depression
Node 2
Mean 37.306
Std. Dev. 5.436
n 402
% 39.2
Predicted 37.306
Self-perceived health - us version
Improvement=2.262
Activities requiring a moderate level of energy
Improvement=1.281
Fair; Excellent and Very good; Good
Node 3
Mean 32.651
Std. Dev. 6.094
n 350
% 34.1
Predicted 32.651
Poor
Node 4
Mean 28.791
Std. Dev. 5.843
n 273
% 26.6
Predicted 28.791
More than once a week
Node 5
Mean 38.723
Std. Dev. 5.011
n 249
% 24.3
Predicted 38.723
Once a week; One to three times a month; Hardly ever, or never
Node 6
Mean 35.000
Std. Dev. 5.325
n 153
% 14.9
Predicted 35.000
Activities requiring a moderate level of energy
Improvement=0.791
Activities requiring a moderate level of energy
Improvement=0.354
More than once a week
Node 7
Mean 34.010
Std. Dev. 5.629
n 197
% 19.2
Predicted 34.010
Once a week; One to three times a month; Hardly ever, or never
Node 8
Mean 30.902
Std. Dev. 6.239
n 153
% 14.9
Predicted 30.902
More than once a week; One to three times a month
Node 9
Mean 30.074
Std. Dev. 5.989
n 122
% 11.9
Predicted 30.074
Once a week; Hardly ever, or never
Node 10
Mean 27.755
Std. Dev. 5.527
n 151
% 14.7
Predicted 27.755
Education level
Improvement=0.284
First stage of tertiary; Upper secondary; Post-secondary non-tertiary
Node 11
Mean 34.864
Std. Dev. 5.146
n 132
% 12.9
Predicted 34.864
Low secondary; Primary; Second stage of tertiary education; None or pre-primary
Node 12
Mean 32.277
Std. Dev. 6.186
n 65
% 6.3
Predicted 32.277
Age
Improvement=0.238
<= 65.5
Node 13
Mean 36.013
Std. Dev. 4.306
n 77
% 7.5
Predicted 36.013
> 65.5
Node 14
Mean 33.255
Std. Dev. 5.800
n 55
% 5.4
Predicted 33.255

Figure S1. Classification and Regression Tree model for CASP Index for Quality of Life (Model without depth limitation).

Table S1. Descriptive analysis of people with all over pain.

		Median	IQR
	Age (Years)	62	15
	Height (cm)	167	10
	Weight (kg)	76	21
	CASP Index for QoL (12-48)	33	9
	Depression Scale (0-12)	4	14
		**n**	**(%)**
**Sex**	Women	750	(73%)
	Men	275	(27%)
**BMI**	Underweight	28	(3%)
	Normal	293	(30%)
	Overweight	371	(38%)
	Obese	285	(29%)
**Education Level**	None or pre-primary	30	(3%)
	Primary	180	(18%)
	Lower secondary	194	(19%)
	Upper secondary	414	(41%)
	Post-secondary non-tertiary	39	(4%)
	First stage of tertiary education	159	(16%)
	Second stage of tertiary education	6	(1%)
	Still in school	1	(0%)
**Level of Pain**	Mild	129	(13%)
	Moderate	520	(51%)
	Severe	374	(37%)
**Smoke at the present time**	No	261	(62%)

	Yes	161	(38%)
**Depression**	No	400	(40%)
	Yes	603	(60%)
**Self-perceived health**	Very good and Excellent	37	(4%)
	Good	215	(21%)
	Fair	433	(42%)
	Poor	339	(33%)
**Moderate PAF**	Hardly ever, or never	255	(53%)
	One to three times a month	82	(14%)
	Once a week	141	(8%)
	More than once a week	546	(25%)
**Vigorous PAF**	Hardly ever, or never	588	(58%)
	One to three times a month	82	(8%)
	Once a week	119	(12%)
	More than once a week	234	(23%)
**QoL Categorical**	Low	601	(59%)
	Moderate	106	(10%)
	High	119	(12%)
	Very High	199	(19%)

BMI (Body max index); CASP (Control, Autonomy, Pleasure and Self-Realization); QoL (Quality of Life); CASP Index for QoL (Higher score indicate a higher QoL); Depression Scale (Higher score indicate a higher degree of depression); n (number); % (Percentage); IQR (Interquartile Range); PAF (Physical Activity Frequency).

Table S2. Prevalence of Quality of Life according to moderate and vigorous physical activity frequency.

Variables	Quality of Life Categorical											
Moderate PAF	Low (A)		Moderate (B)		High (C)		Very high (D)		X2	df	p-value	V
	n	%	n	%	n	%	n	%				
More than once a week	249	41.4%	66	62.3%	77	64.7%	154	77.8%				
Once a week	103	17.1%	11	10.4%	15	12.6%	12	6.1%	97.6	9	<.001	.178
One to three times a month	52	8.7%	11	10.4%	8	6.7%	11	5.6%				

Hardly ever, or never	197	32.8%	18	17.0%	19	16.0%	21	10.6%				
Proportions' differences post hoc												
More than once a week			A(p<.001)***		A(p<.001)***		A(p<.001)*** B(p=.024)*					
Once a week	D(p=.001)**											
One to three times a month												
Hardly ever, or never	B(p=.007)** C(p=.002)** D(p<.001)***											

Variables	Quality of Life Categorical											
Vigorous PAF	Low (A)		Moderate (B)		High (C)		Very high (D)		X2	df	p-value	V
	n	%	n	%	n	%	n	%				
More than once a week	98	16.3%	26	24.5%	40	33.9%	70	35.2%	56.2	9	<.001	.135
Once a week	58	9.7%	20	18.9%	14	11.9%	27	13.6%				
One to three times a month	53	8.8%	5	4.7%	8	6.8%	16	8.0%				
Hardly ever, or never	391	65.2%	55	51.9%	56	47.5%	86	43.2%				
Proportions' differences post hoc												
More than once a week						A(p<.001)***		A(p<.001)***				
Once a week				A(p=.032)*								
One to three times a month												
Hardly ever, or never		C(p=.002)**										

p (p-value from pairwise z-test for independent proportions); * (p<0.05); *** (p<0.001); X2 (Chi-Square); df (Degree freedom); V (V's Cramer coefficients); PAF (Physical Activity Frequency)
